# Investigating the involvement of potato (*Solanum tuberosum* L.) *StPHR1* gene in the combined stress response to phosphorus deficiency and aluminum toxicity

**DOI:** 10.3389/fpls.2024.1413755

**Published:** 2024-06-21

**Authors:** Feng Zhang, Wenlun Wang, Anping Yuan, Qiong Li, Moli Chu, Sixia Jiang, Yanlin An

**Affiliations:** ^1^ Department of Food Science and Engineering, Moutai Institute, Luban Street, Renhuai, Guizhou, China; ^2^ Department of Brewing Engineering, Moutai Institute, Luban Street, Renhuai, Guizhou, China; ^3^ Anhui Provincial Key Laboratory of the Conservation and Exploitation of Biological Resources/College of Life Sciences, Anhui Normal University, Wuhu, Anhui, China

**Keywords:** Al toxicity, gene, Pi deficiency, potato, *StPHR1*

## Abstract

Phosphorus deficiency and aluminum toxicity in acidic soils are important factors that limit crop yield. To further explore this issue, we identified 18 members of the *StPHR* gene family in the potato genome in this study. Through bioinformatics analysis, we found that the *StPHR1* gene, an important member of this family, exhibited high expression levels in potato roots, particularly under conditions of phosphorus deficiency and aluminum toxicity stress. This suggested that the *StPHR1* gene may play a crucial regulatory role in potato’s resistance to phosphorus deficiency and aluminum toxicity. To validate this hypothesis, we conducted a series of experiments on the *StPHR1* gene, including subcellular localization, GUS staining for tissue expression, heterologous overexpression, yeast two-hybrid hybridization, and bimolecular fluorescence complementation (BiFC). The results demonstrated that the *StPHR1* gene is highly conserved in plants and is localized in the nucleus of potato cells. The heterologous overexpression of the gene in Arabidopsis plants resulted in a growth phenotype that exhibited resistance to both aluminum toxicity and phosphorus deficiency. Moreover, the heterologous overexpressing plants showed reduced aluminum content in the root system compared to the control group. Furthermore, we also identified an interaction between *StPHR1* and *StALMT6*. These results highlight the potential application of regulating the expression of the *StPHR1* gene in potato production to enhance its adaptation to the dual stress of phosphorus deficiency and high aluminum toxicity in acidic soils.

## 1 Introduction

Acidified soils account for about 30–40% of the world’s arable soils globally, and even up to 70% in some parts of the subtropics (Kochian et al., 2015). These soils have two major limiting factors for plant growth, aluminum toxicity and phosphorus deficiency (Chen et al., 2022, Chen et al, 2022; Ur Rahman et al., 2024). Under acidic soil conditions, especially with a pH below 5, aluminum ions and soil constituents such as iron and its oxides bind more strongly to soil phosphorus, making it less accessible and available to plants (Kochian et al., 2004; Sade et al., 2016). For example, Liang et al. analyzed the actual soil from the surface to the deeper layers in the Boro area of Guangdong Province, and the experimental data intuitively reflected that the deeper the soil layer, the lower the pH, the higher the content of aluminum ions, while the content of phosphorus is getting lower and lower (Liang et al., 2013). Consequently, aluminum toxicity and phosphorus deficiency stress often coexist in acidic environments, imposing interactive effects on plant growth and development (Jemo et al., 2007).

It was found that increasing the supply of effective phosphorus could alleviate the toxic effects of aluminum toxicity on some plants, such as sour grapefruit (Jiang et al., 2009), maize (Gaume et al., 2001) and huckleberry (Sun et al., 2008a). This is explained by the fact that inorganic phosphorus can directly precipitate with aluminum ions to form complexes that are non-toxic to plants, which in turn reduces the toxicity of aluminum to plants. In addition, some studies have explained that when effective phosphorus is supplied, some of it reacts with aluminum ions in the soil to produce phosphorus-aluminum complex precipitates that are not hazardous to plants, and some of it prevents the transport of aluminum from roots to the ground, thus improving the aluminum tolerance of plants (Pierre and Stuart, 1933; Yang et al., 2011). Later, in soybean phosphorus aluminum experiments, it was found that there may be antagonism between soybean-induced root secretion of organic acids and “aluminum toxicity and phosphorus deficiency” conditions (Liao et al., 2006; Chen et al., 2010). Aluminum toxicity and phosphorus deficiency interactions resulted in fewer organic acids being secreted compared to single factor (aluminum toxicity or phosphorus deficiency) stresses (Gomez-Zepeda et al., 2021). Phosphorus aluminum interactions had a significant effect on soybean growth and “aluminum toxicity and phosphorus deficiency” stresses.

Phosphorus-aluminum interaction has a regulating and controlling effect on soybean growth and on the mechanism of organic acid secretion induced by aluminum toxicity and phosphorus deficiency (Liao et al., 2006). Short-term phosphorus deficiency in plants induces citric acid secretion from soybean roots, while prolonged phosphorus deficiency significantly reduces citric acid secretion. Both aluminum toxicity and phosphorus deficiency reduce the dry weight of grapefruit roots and aboveground. Increased phosphorus application promotes aluminum fixation in citrus roots and increases the phosphorus content of roots, stems, and leaves, which can reduce the aboveground aluminum content and thus alleviate the growth suppression caused by aluminum stress (Jiang et al., 2009). In buckwheat, there is a significant interaction between phosphorus and aluminum elements in the root tips, and the phosphorus content in the root tips of plants is closely related to aluminum fixation and detoxification of aluminum (Zheng et al., 2005). The application of a certain amount of phosphorus can greatly increase the biomass and root-crown ratio of buckwheat, significantly reduce the content of exchangeable aluminum in the inter-root medium under aluminum stress, enhance the activity of peroxidase in the inter-root soil, and alleviate the growth suppression of aluminum toxicity on buckwheat (Che et al., 2018). Increased application of exogenous phosphorus can alleviate the peroxidation of rice membrane lipids induced by aluminum toxicity, reduce the accumulation of reactive oxygen species in the rice body, and alleviate the damage caused by aluminum toxicity to rice roots and leaves (Huang et al., 2013).

Elemental phosphorus is an essential bulk nutrient for plants and plays a crucial role in their growth and development (Yang et al., 2024). However, plants often face Pi starvation due to the limited availability of soluble Pi in the soil, as it tends to bind with organic and inorganic compounds to form insoluble complexes (Yang and Finnegan, 2010). In response to phosphorus deficiency, plants activate various biochemical, physiological, and developmental responses (Vance et al., 2003; Paz-Ares et al., 2022), collectively known as Pi starvation responses (PSRs) (Yang et al., 2024). These responses aim to improve phosphorus utilization efficiency and include both local and systemic mechanisms. Systemic responses involve the regulation of Pi uptake, translocation, and recycling, primarily influenced by the Pi concentration within the plant (Thibaud et al., 2010). Transcriptional regulation plays a crucial role in the response of plants to phosphorus (Pi) starvation, and one key transcription factor involved in this process is *PHR1* (Sega and Pacak, 2019). *PHR1* belongs to the MYB-CC family and contains a CC structural domain, with Arabidopsis having 15 homologous genes in this family (Rubio et al., 2001; Sun et al., 2016). The identification of a *phr1* mutant through EMS mutagenesis revealed that a reporter gene specifically responsive to Pi starvation had a weakened response under such conditions (Rubio et al., 2001). Further investigation demonstrated that *PHR1* binds to a palindrome sequence, known as the PHR1-Binding Sequence (P1BS) or *PHR1*-binding element, present in the promoters of many Pi starvation-induced (PSI) genes, and this sequence serves as a crucial cis-regulatory motif in Pi starvation signaling (Bustos et al., 2010). Additional studies revealed that the miR399/PHO2 pathway acts downstream of the *PHR1* phosphorus signaling network, with PHO2 encoding the E2 splicase, which is regulated by the Pi-dependent miR399 (Bari et al., 2006). Moreover, overexpression of *PHR1* in Arabidopsis resulted in increased expression of *PSI* genes and a significant increase in aboveground phosphorus content (Nilsson et al., 2007), highlighting the central role of *PHR1* as a transcription factor in the regulatory network of the phosphorus starvation signaling response pathway. Furthermore, studies have shown that *PHR1* is partially redundant with *PHL1* (PHR1-like 1) and *PHL2* in controlling physiological and molecular responses to Pi starvation.

Proteins containing SPX domains play a crucial role in maintaining phosphorus homeostasis in plants during periods of phosphorus starvation (Secco et al., 2012). These proteins are divided into four families based on the presence of additional domains in their structures: SPX, SPX-EXS, SPX-MFS, and SPX-RING families (Secco et al., 2012). Proteins that solely contain the SPX domain are referred to as SPX proteins. In *Arabidopsis* and rice, the SPX family consists of four and six members, respectively (Duan et al., 2008; Wang et al., 2009). Transcriptional and histochemical analyses have demonstrated that all *SPX* genes, except for *AtSPX4* and *OsSPX4*, are significantly up-regulated in response to phosphorus starvation, both in the roots and aboveground (Duan et al., 2008; Wang et al., 2009). Studies have revealed that SPX1 functions as a repressor of the phosphorus starvation response in *Arabidopsis*, primarily by negatively regulating the expression of *PHR1* (Puga et al., 2014). When phosphorus levels are sufficient, SPX1 binds to PHR1 with high affinity, thereby inhibiting PHR1’s ability to bind to downstream PSI targets and reducing *PHR1* activity. However, under conditions of phosphorus starvation, *SPX1* no longer binds to *PHR1*, allowing *PHR1* to freely bind to downstream *PSI* genes and enhance their expression levels in response to phosphorus starvation (Puga et al., 2014). Interestingly, it was recently found that the key plant aluminum toxicity genes *STOP1* (Huang, 2021; Paz-Ares et al., 2022), *ALS3/STAR1* (Che et al., 2018), and *ALMT1* (Delhaize et al., 2009; Chen et al., 2017; Paz-Ares et al., 2022) all play important roles in the phosphorus deficiency response and stress. However, there are no published studies on key genes of the phosphorus deficiency response in response to aluminum toxicity.

Research on the response of potato to phosphorus deficiency and aluminum toxicity stress has not been extensively reported in the literature. Currently, the focus of aluminum toxicity research in potato is mainly on the screening and evaluation of tolerant varieties, as well as the determination of physiological indicators (Tabaldi et al., 2009). Our previous studies identified the potato St*ALMT* gene family and functionally analyzed its members *StALMT6/10* in response to aluminum toxicity (Zhang et al., 2023). The primary aim of this study was to characterize and analyze the *StPHR1* gene in potato. Our focus was to understand the evolutionary relationship, spatial structure, subcellular localization, tissue-specific expression pattern, and functional role of the *StPHR1* gene. Additionally, we investigated the response mechanism of the *StPHR1* gene under the dual stress conditions of phosphorus deficiency and aluminum toxicity. Furthermore, we explored the interrelationship between the *StALMT6* gene, which encodes a malate secretion transporter in potatoes, and the *StPHR1* gene in response to the combined stresses of phosphorus deficiency and aluminum toxicity.

## 2 Materials and methods

### 2.1 Materials and conditions for plant culture

The potato cultivar “Qingshu No. 9” was used in this study and was obtained from the Institute of Vegetable and Flower Research, Chinese Academy of Agricultural Sciences (CAAS). Following germination in a 25°C environment, the original stock was transferred to a 1/6 potato MS medium containing vitamin (Coolaber) unbuffered solution at an initial pH of 5.5. Potatoes were grown in an incubation room at 25°C with a photoperiod of 12 h light/12 h dark.

The wild-type (WT) of *Arabidopsis thaliana* (Col-0) was obtained from the College of Life Sciences, Anhui Normal University. In the growth experiments, WT seeds were sterilized with 75% ethanol for 3 minutes, washed four times with double-distilled water (DDW), and then sown on 1/2 MS medium containing 1% sucrose and agar (Aldrich-Sigma). The phenotypic methods for experimental growth of Arabidopsis with phosphorus deficiency and aluminum toxicity are described in our previous study (Zhang et al., 2019). Solid medium plates were incubated at 4 °C in the dark for 2 days and then placed vertically. On day 9 of poste mergence incubation, seedlings were imaged using Image J software (http://rsb.info.nih.gov/ij/) to measure primary root length or collected to weigh biomass.

### 2.2 The PHR gene family was subjected to characterization and analysis

The protein sequence of the potato reference genome “*Solanum tuberosum* v6.1” was obtained from the Phytozome 13 database (https://phytozome-next.jgi.doe.gov/). Subsequently, members of the *PHR* gene family in potato were identified through gene annotation and Blastp homology matching. The subcellular localization of the PHR family proteins was predicted using the WoLF PSORT (https://www.genscript.com/wolf-psort.html) online tool.

### 2.3 Constructing a phylogenetic tree for the *StPHR* gene family in potato

The sequences of related homologous proteins of potato *StPHR* family genes were obtained by searching LHEQLE and SHAQKYF sequences in the Phytozome 13 *Solanum tuberosum* v6.1 database. These sequences, together with the sequences of PHR family proteins of Arabidopsis (*Arabidopsis thaliana*) and tomato (*Solanum lycopersicum*), were used to construct a phylogenetic tree of potato StPHRs family proteins using MEGA7.0 (NJ method, with a Rootstrap value of 1000). The spatial structure of StPHR1 was predicted using DMFold (https://zhanggroup.org/DMFold/) and Phyre2 (http://www.sbg.bio.ic.ac.uk/~phyre2/html/page.cgi?id=index), respectively.

### 2.4 Analysis of the potato PHR gene family by RT-PCR and Q-PCR

Total RNA was extracted and collected from potato and *Arabidopsis thaliana* using TRIzol reagent. Complementary cDNAs were then synthesized with M-MLV reverse transcriptase from Promega. Primers for this study involving the gene were designed using Primer Premier 5.0 software (**Supplementary Table S2**). The internal reference gene used in the PCR assay analysis was *ACTIN* gen (Soltu.DM.03G011750.2). All primers used are listed in **Supplementary Table S1**. Three biological replicates were performed for each experimental sample, and differential expression was calculated using the 2 (-Delta C(T)) method (Livak and Schmittgen, 2001).

### 2.5 Subcellular localization of *StPHR1*

Using recombinant DNA technology, the CDS sequence of StPHR1 (Soltu.DM.05G026640) was inserted into the downstream sequence of the GFP protein encoded by pBWA(V)HS - GLosgfp vector to form the fusion expression vector pBWA(V)HS-StPHR1-GLosgfp, which was transformed into native tobacco leaves through PEG mediation for the analysis of subcellular localization.

### 2.6 Full-length CDS sequence cloning of the potato *StPHR1* gene

The roots of Qingshu No. 9 were selected as the material for RNA extraction. Total RNA was extracted using an extraction kit (Tiangen Biochemical Technology (Beijing) Co., Ltd.). Subsequently, cDNA was synthesized through reverse transcription. For PCR amplification of the *PHR1* gene’s CDS sequence, the primers St-*PHR1*-F3 and St-*PHR1*-R3 from **Supplementary Table S2** were utilized. The amplification products were separated by 0.8% Agarose gel electrophoresis. The concentration of the recovered CDS fragments was determined using a DNA Gel Extraction Kit. The recovered product was then subjected to poly A tailing with Taq enzyme, followed by ligation into the pMD 19-T vector and transformation into *E. coli* DH 5α receptor cells. The cells were cultured on screening medium containing antibiotics, and subsequently, monoclonal colonies were selected for PCR, calibration, and sequencing verification.

### 2.7 The construction of overexpression vectors and transformation of *Arabidopsis thaliana*

To construct an overexpression vector for the *StPHR1* transgene, the coding sequence (CDS) of StPHR1 was amplified from the cDNA of Qingshu No. 9. During transformation in Arabidopsis, the CDS sequence of *StPHR1* was ligated to the EcoR I/Xba I site of the plant expression vector pCAMBIA1300 to obtain the plant overexpression vector pCAMBIA1300–35S-*StPHR1*. The overexpression vector was then transferred into Agrobacterium tumefaciens strain GV3101, and Arabidopsis WT plants were transformed using the flower dip method (Clough and Bent, 1998).

### 2.8 Staining of aluminum ions by fluorescence

A total of 6.0448 grams of Morin, was weighed and dissolved in 5 mL of methanol to make a 4 mM master batch. This master batch was stored in a refrigerator at 4°C for later use. To prepare the working concentration, 1 mL of the 4 mM mother liquor was pipetted and mixed with 39 mL of DDW, resulting in a final concentration of 0.1 mM. This working solution was also stored in the refrigerator at 4 °C. For the experiment, plates containing WT and overexpression Arabidopsis seedlings, which had been grown vertically for approximately 5 days in a plant light incubator, were unsealed. From these plates, healthy WT and overexpression Arabidopsis seedlings with uniform root length and good growth were carefully selected. These selected seedlings were then transferred to separate plates, with one plate serving as the control and the other treated with 250μM AlCl_3_. Transfer was done using sterilized toothpicks. After transfer, the plates were sealed with 3 M adhesive tape and placed back in the light incubator for 12 h. Following the incubation period, the control and treated plates were removed from the incubator. The plates were sealed again with 3 M adhesive tape and placed back in the incubator. Seedlings were carefully removed from the control and treated plates and washed three times with DDW. After washing, the seedlings were immersed in a staining solution, wrapped in tin foil, and incubated at 22°C for 0.5 h. Once the staining was complete, the seedlings were washed three times with DDW. Finally, the seedlings were prepared and photographed under a microscope.

### 2.9 The determination of aluminum content in *Arabidopsis thaliana*

The Arabidopsis seedlings were cultured on 1/2 MS solid medium for 7 days and then transferred to 1/2 MS (pH 5.8) nutrient solution for duration of 2 weeks. Subsequently, they were transferred to a freshly prepared homogenous nutrient solution, with or without the presence of aluminum, for treatment. The concentration of aluminum in the nutrient solution was set at 250 µM, and the treatment duration lasted for 72 h. Root samples were collected, carefully dried on blotting paper, weighed, and then placed into 15 mL centrifuge tubes. To initiate digestion, 500 µL of concentrated nitric acid was added to the tubes, and the samples were heated at 100°C for approximately 3 h using an ablator. The centrifuge tubes were further supplemented with an additional 500 µL of concentrated nitric acid, followed by digestion at 100°C for about 3 h. When digestion was completed, the samples were centrifuged and the collected supernatant was transferred to a clean tube for further analysis. To reach a final volume of 10 mL, DDW was added to the tube, and subsequent centrifugation was performed at 3000 rpm for 5 minutes. A 500 µL aliquot was taken from the resulting supernatant and diluted 2-fold before determining the aluminum content using Inductively Coupled Plasma Mass Spectrometry (ICP-MS) according to the method described by our previous study (Zhang et al., 2019).

### 2.10 Measurement of inorganic phosphorus content

The plant material to be tested (according to experimental requirements) can be divided into above-ground and below-ground plants. The material is washed three times with DDW and any water stains on the surface are absorbed using absorbent paper. The fresh weight of the plant material is then measured on a balance. To prepare the grinding solution, DDW is added to the sample in a ratio of 1:10 in a mortar and pestle, and the mixture is ground until homogeneous. The grinding solution is then transferred to a centrifuge tube and placed on ice. The tube is centrifuged at 10,000 rpm for 5 minutes at 4°C using a refrigerated centrifuge. The supernatant is carefully transferred to a new centrifuge tube for further chemical reaction to determine phosphorus content. In a reaction tube, 4.75 mL of DDW is added, followed by 250 μL of the supernatant and 200 μL of 10% ascorbic acid solution (used as is). The mixture is shaken for 20 seconds until well-mixed. Then, 400 μL of molybdate solution is quickly added and mixed thoroughly. The reaction is left for 10 minutes. Next, 1 mL of the reaction solution is taken in a cuvette and the OD value at 690 nm is measured using a spectrophotometer, with DDW as a blank control. The obtained data are recorded. To determine the OD value at 690 nm of the reaction sample using NaH_2_PO_4_ as the Pi source, the same experimental method is followed.

### 2.11 Statistical analysis

In order to ensure the accuracy and reliability of the findings, at least three independent replications were performed in each experiment. This rigorous methodology ensured that we obtained robust and consistent data. To assess the statistical significance of our findings, the widely used Student’s t-test was employed with a significance threshold set at P<0.05.

## 3 Results

### 3.1 Phylogenetic analysis and subcellular localization prediction of PHR protein family

**Figure 1:**
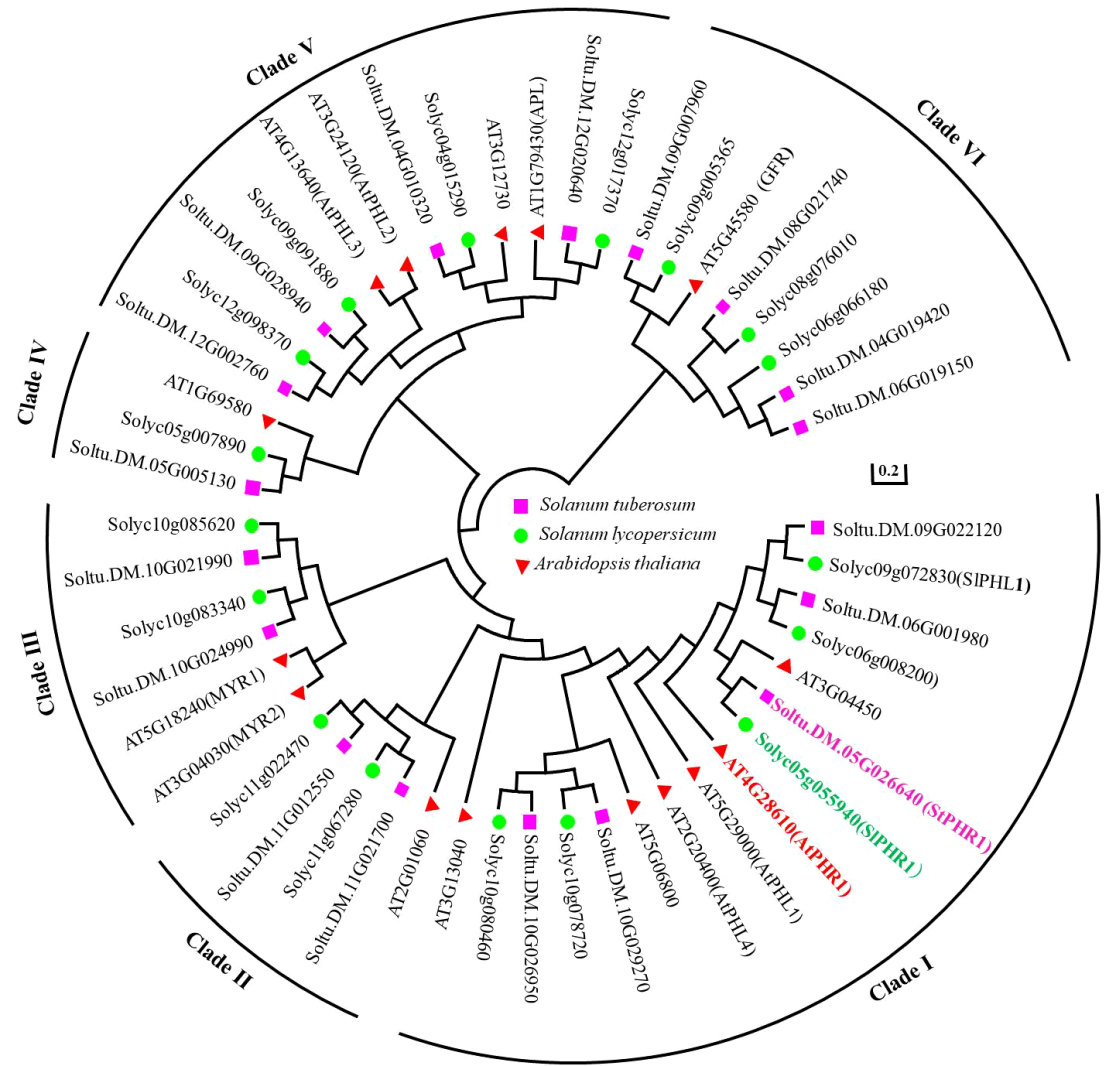
Phylogenetic tree analysis of PHR protein family of Arabidopsis, potato and tomato by amino acid homologous sequence comparison. The protein sequences of PHR protein family from Arabidopsis, potato and tomato were subjected to homologous multiple sequence alignment using MEGA 7.0 software, and phylogenetic trees were constructed using the neighbor-joining (NJ) method with a Bootstrap value of 1,000.

By searching the Phytozome 13 *Solanum tuberosum* v6.1 database, the structural domains of potato StPHR proteins and their homologous proteins were obtained using the LHEQLE (PF14379) and SHAQKYF (Xu et al., 2018) sequences. A total of 18 members were identified after removing duplicate sequences and arranging *StPHR* based on their chromosomal positions in potato. To predict their subcellular localization, the WoLF PSORT online website (https://www.genscript.com/wolf-psort.html) was utilized, providing information on the subcellular distribution of *StPHR1* gene family (**Supplementary Table S1**). Furthermore, for a comprehensive analysis of the evolutionary relationships within the PHR gene family, a comparison was made among 18 potato StPHR protein sequences, 16 tomato SlPHR protein sequences, and 15 *Arabidopsis* AtPHR protein sequences, resulting in the construction of a phylogenetic tree (**Figure 1**). The findings revealed that the PHR family proteins from Arabidopsis, potatoes, and tomatoes can be classified into six distinct types, with StPHR1 belonging to Clade I (Class I).

### 3.2 Conservation of *PHR1* proteins in potato, *Arabidopsis*, and tomato suggests similar functions in response to phosphorus deficiency and aluminum toxicity

**Figure 2:**
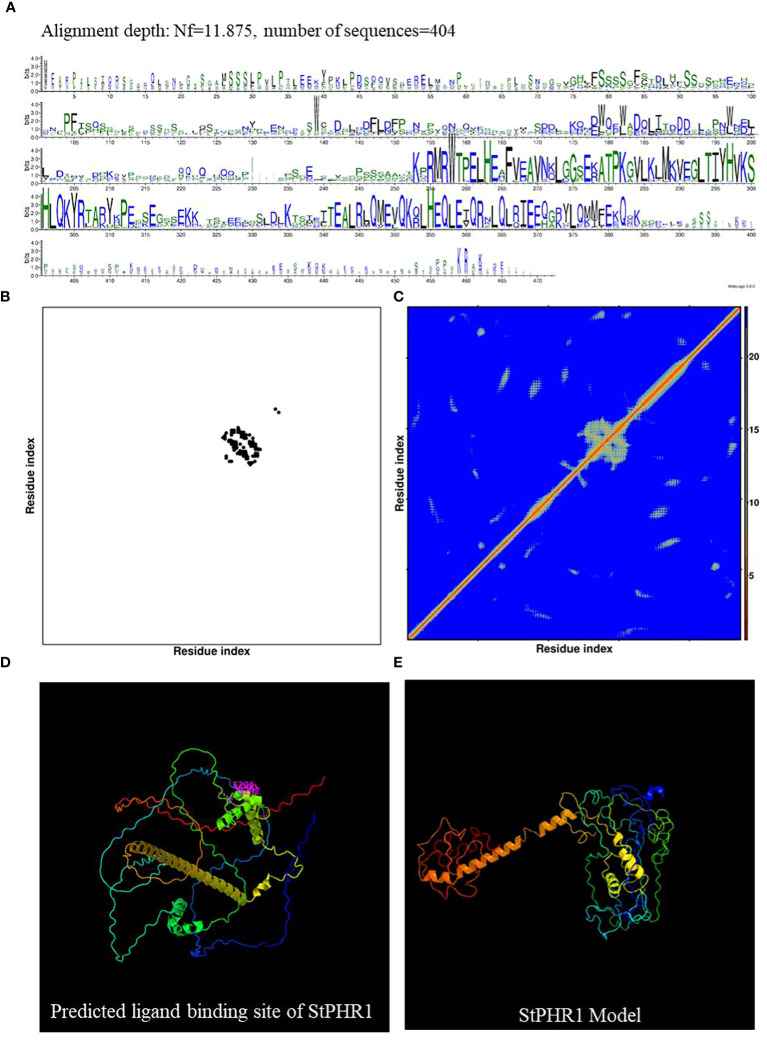
DMFold predicts the spatial structure of StPHR1 protein. **(A)** Multiple sequence alignments of amino acids of StPHR1 protein; **(B)** Predicted contact map of StPHR1 protein; **(C)** Predicted distance map of StPHR1 protein; **(D)** Predicted ligand binding sites of StPHR1 protein; **(E)** Predicted final model of StPHR1 protein.

Potato (*Solanum tuberosum*) and tomato (*Solanum lycopersicum*), both members of the *Solanaceae* family, were the subjects of this study aimed at investigating the functional role of the potato *StPHR1* (Soltu.DM.05G026640) gene in response to phosphorus deficiency and aluminum toxicity. To establish a solid theoretical foundation for this study, we referred to the gene and protein functional information of *Arabidopsis* (*Arabidopsis thaliana*) *AtPHR1* (AT4G28610) and tomato *SlPHR1* (Solyc05g055940) (Nilsson et al., 2007; Liao et al., 2022). We comparatively analyzed the full-length amino acid sequences of AtPHR1, SlPHR1, and StPHR1, revealing a high degree of homology among these PHR1 proteins. Conserved MYB and coiled-coil (CC) sequence regions were observed, indicating the evolutionary conservation of the PHR1 protein (**Supplementary Figure S1**). To further explore the StPHR1 protein, we predicted its spatial structure using DMFold and Phyre2 online software, respectively (**Figure 2**). The results demonstrated that the structure of StPHR1 closely resembled that of SlPHR1 and AtPHR1 (**Figures 2C, D**; **Supplementary Figure S2**), suggesting that StPHR1 proteins may possess similar functional and structural features across different species. These findings provide valuable insights for further investigation into the role of the potato StPHR1 protein in response to phosphorus deficiency and aluminum toxicity.

### 3.3 *StPHR1* is localized in the nucleus and the root

**Figure 3:**
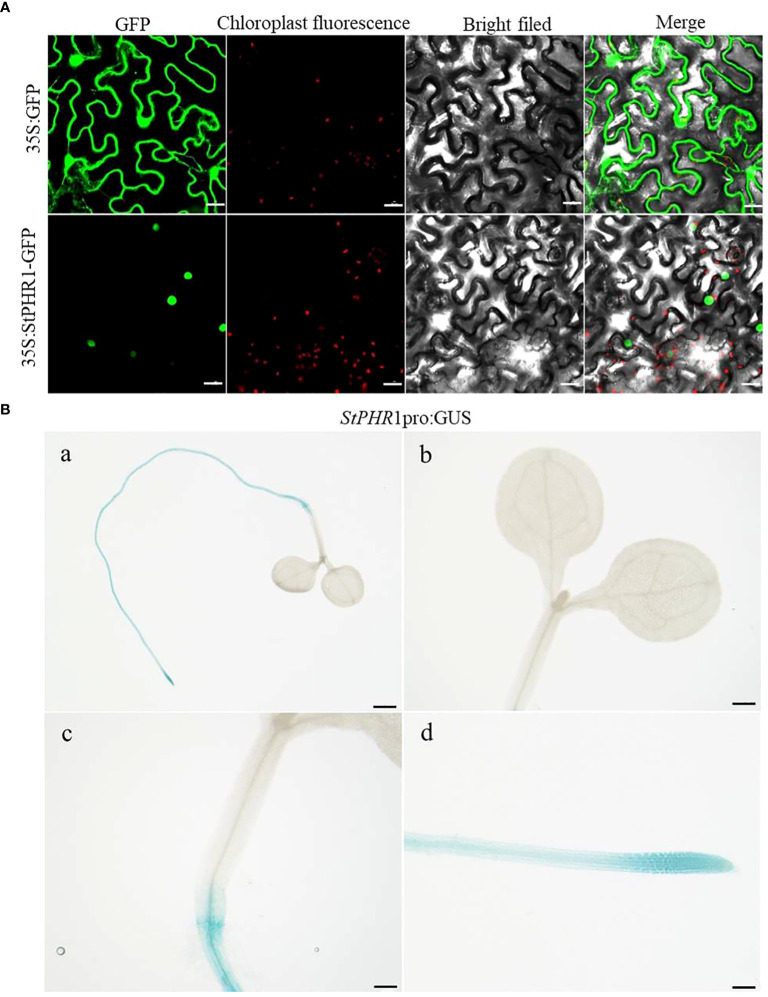
Subcellular localization and tissue-level expression of *StPHR1*. **(A)** The subcellular localization of potato *StPHR* was examined. The figures from left to right represent GFP channels, chloroplast fluorescence, bright field image, and merged image. The scale bar length is 200 µm. **(B)** GUS staining was performed on transgenic seedlings expressing *StPHR1* pro:GUS construct, which were grown in petri dishes for 5 days. Scale in a is 1 mm. Scale in b–d is 500 μm.

For this study, we constructed the pBWA(V)HS-35S-StPHR1-GFP subcellular localization vector and transfected it into native tobacco cells to observe the localization of StPHR1. The localization of the empty 35S:GFP vector was also compared to that of the pBWA(V)HS-35S-StPHR1-GFP vector containing StPHR1 through microscopic observation and image analysis. The results demonstrated that StPHR1 localized to in the nucleus, in contrast to the empty vector. The nuclear localization of StPHR1 suggests a potential role in gene expression regulation, as the nucleus serves as the core component carrying genetic information and regulatory mechanisms within the cell (**Figure 3A**). Previous research has indicated the involvement of the PHR1 and protein families of *Arabidopsis* and tomato in crucial physiological processes such as phosphate uptake and transport in plants (Sega and Pacak, 2019; Isidra-Arellano et al., 2021).

To further investigate the expression pattern of the *StPHR1* gene in various tissues, we utilized *StPHR1* pro:GUS transgenic seedlings in this study and conducted GUS staining analysis. The results revealed a predominant expression of the *StPHR1* gene in root tissues, with the highest expression level observed in the root tip region (**Figure 3B**). These findings shed light on the functional significance of StPHR1 and lay the groundwork for future investigations into its precise role in potato physiology. Understanding the subcellular and tissue localization of StPHR1 may facilitate the development of strategies to enhance phosphate uptake efficiency and improve aluminum stress tolerance in potato crops. Further studies are warranted to unravel the intricate molecular mechanisms underlying StPHR1-mediated regulation, which will provide valuable insights into the genetic and molecular basis of plant nutrient homeostasis and stress response.

### 3.4 Up-regulation of *StPHR1* gene expression in response to aluminum toxicity and phosphorus deficiency in potato seedlings

**Figure 4:**
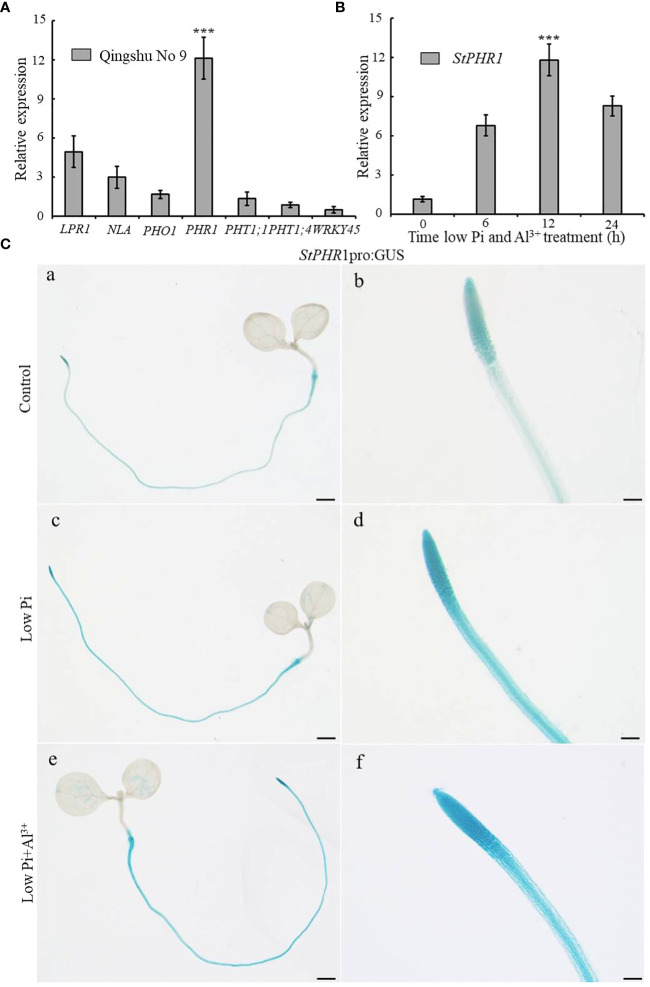
Changes in the expression of phosphorus-related genes of potato after being subjected to aluminum toxicity. **(A)** The relative expression of *StLPR1* (Soltu.DM.05G004660), *StNLA* (Soltu.DM.09G023400), *StPHO1* (Soltu.DM.09G026960), *StPHR1* (Soltu.DM.05G026640)*, StPHT1;1* (Soltu.DM.09G026670), *StPHT1;4* (Soltu.DM.09G026680), *and StWRKY45* (Soltu.DM.05G012130) genes in potato roots was assessed after a 12-hour treatment with normal and aluminum ions; **(B)** The expression of the *StPHR1* gene in Qingshu No. 9 was examined following treatments with low phosphorus and aluminum ions at different time points (0 h, 6 h, 12 h, and 24 h); *ACTIN* (Soltu.DM.03G011750.2) was used as an internal standard. Statistical results of the data. *** indicates significant difference P<0.001. **(C)** *StPHR1* pro:GUS transgenic seedlings after different treatments for their GUS staining. Staining results of transgenic seedlings (grown in Petri dishes for 5 days) after 12 h of treatment with or without low phosphorus, low phosphorus and 250 µM Al^3+^, respectively. Scale in a, c, and e is 1 mm. Scale in b, d, and f is 500 μm.

In order to investigate the potential role of low-phosphorus genes in potatoes under aluminum toxicity, this study examined the expression levels of several low-phosphorus-related genes, including *StLPR1*, *StNLA*, *StPHO1*, *StPHR1*, *StPHT1;1*, *StPHT1;4*, and *StWRKY45* genes in the roots of potato seedlings exposed to aluminum stress. The results demonstrated a significant up-regulation of the *StPHR1* gene, with a 12.1-fold increase compared to the control group (**Figure 4A**). To further substantiate the distinct alterations in *StPHR1* gene expression induced by aluminum toxicity, we evaluated the expression levels of the *StPHR1* gene in Qingshu No. 9 under varying durations of low phosphorus and aluminum toxicity stresses through q-PCR experiments. Our analysis revealed a marked up-regulation of the *StPHR1* gene, with expression levels escalating by 6.0-fold, 10.4-fold, and 7.3-fold at 6 h, 12 h, and 24 h of treatment, respectively. Notably, the most significant elevation in expression was observed at the 12 h treatment mark (**Figure 4B**).

Furthermore, to validate the changes in *StPHR1* gene expression at the tissue level and confirm the q-PCR results, we utilized *StPHR1* pro:GUS seedlings cultured on 1/2 MS medium for approximately 5 days. Subsequently, these seedlings were subjected to 12 h of stress treatment on low phosphorus and low phosphorus plus aluminum ion medium. GUS staining was performed, and the staining results were observed. The findings revealed a deepened color of *StPHR1* pro:GUS staining after the low phosphorus plus aluminum ion stress treatments, particularly indicating enhanced expression in the root, especially in the apical portion (**Figure 4C**). In conclusion, these findings suggest that low-phosphorus genes may play a role in potatoes under aluminum toxicity and phosphorus deficiency, with the up-regulation of *StPHR1* gene expression potentially serving as a key factor.

### 3.5 Overexpression of *StPHR1* enhances tolerance to aluminum toxicity and phosphorus deficiency in *Arabidopsis*

**Figure 5:**
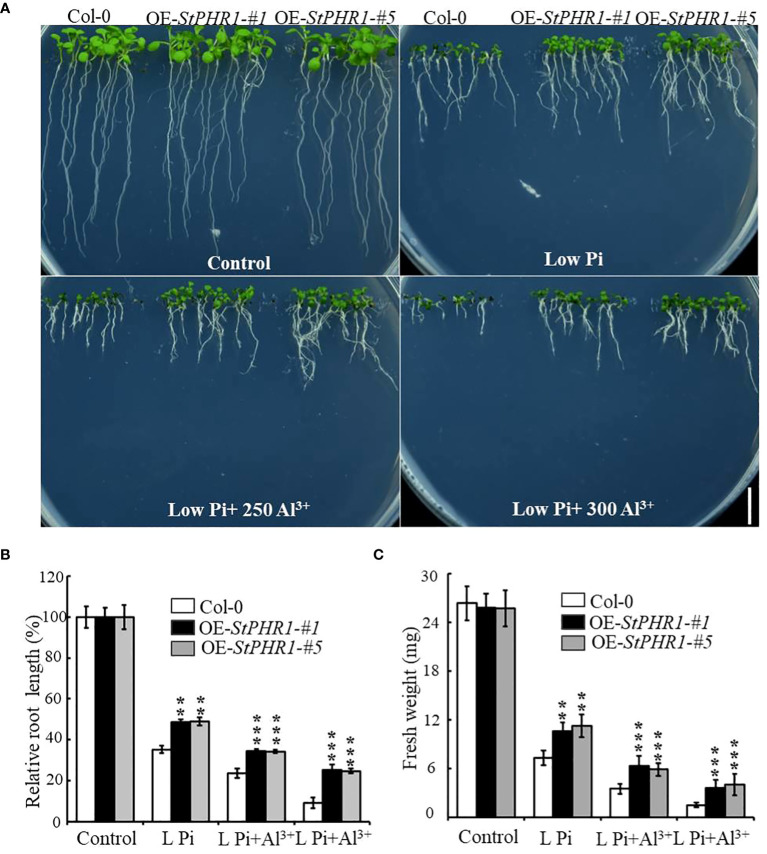
Growth phenotypes of Col-0, OE-*StPHR1-#1*, and OE-*StPHR1-#5* were evaluated under stress treatments involving varying concentrations of aluminum ions, both under normal and low phosphorus conditions. **(A)** Growth phenotypes, biomass relative root length **(B)** and fresh weight **(C)**. Scale bar = 1.0 cm. Statistical results of the data. ** indicates significant difference P<0.01, *** represents P<0.001.

To verify the balancing mechanism of *StPHR1* in the dual stresses of phosphorus deficiency and aluminum toxicity, a 35S-initiated plant binary expression vector containing the *StPHR1* gene was constructed and introduced into Arabidopsis WT (Col-0) plants. Following screening and identification, transgenic lines overexpressing *StPHR1*, designated as OE-*StPHR1-#1* and OE-*StPHR1-#5*, were obtained. Subsequently, Col-0, OE-*StPHR1-#1*, and OE-*StPHR1-#5* were subjected to stress treatments involving different concentrations of aluminum ions under normal and low phosphorus conditions. The results revealed that OE-*StPHR1-#1* and OE-*StPHR1-#5* exhibited growth phenotypes that were resistant to aluminum toxicity compared to Col-0 (**Figure 5A**). Furthermore, biomass analysis confirmed this observation (**Figures 5B, C**). These findings underscore the significance of *StPHR1* overexpression in enhancing plant resilience against aluminum toxicity and phosphorus deficiency. They provide valuable insights into the pivotal role of *StPHR1* in mitigating the dual challenges of phosphorus deficiency and aluminum toxicity, and offer promising avenues for further exploration of plant adaptation strategies under environmental stressors.

### 3.6 Effects of aluminum toxicity and phosphorus deficiency on inorganic phosphorus distribution and aluminum content in plants overexpressing *StPHR1* gene

**Figure 6:**
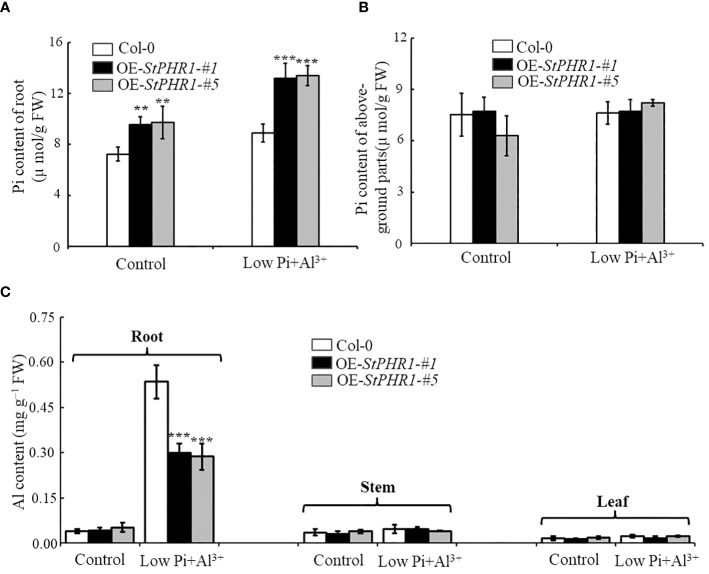
Alterations in the allocation of inorganic phosphorus (Pi) and aluminum content in plants are observed in response to the stress induced by aluminum toxicity and phosphorus deficiency. **(A)** Root Pi content of Col-0, OE-*StPHR1-#1*, and OE-S*tPHR1-#5* after 48 h of treatment with or without low phosphorus (10 µM) and aluminum ions (250 µM); **(B)** After 48 hours of treatment with or without low phosphorus (10 µM) and aluminum ions (250 µM), the Pi content of aboveground parts was measured in Col-0, OE-*StPHR1-#1*, and OE-*StPHR1-#5*; **(C)** Aluminum content of roots, stems and leaves of Qingshu No. 9 after 48 h of treatment with or without low phosphorus (10 µM) and aluminum ions (250 µM). Statistical results of the data. ** indicates significant difference P<0.01, *** represents P<0.001.

To investigate whether the impacts of aluminum toxicity and phosphorus deficiency on plant growth phenotypes are due to variations in inorganic phosphorus distribution or concentration, this study initially employed a molybdate spectrophotometric technique to quantify the phosphorus content in Col-0, OE-*StPHR1-#1*, and OE-*StPHR1-#5*. After subjecting approximately 3-week-old Col-0, OE-*StPHR1-#1*, and OE-*StPHR1-#5* to aluminum ion and low phosphorus stress treatments for 48 hours under standard hydroponic conditions, plants with consistent growth and similar fresh weights were selected to determine the inorganic phosphorus content in roots and above-ground parts. The experimental results showed no significant difference in the inorganic phosphorus content of Col-0, OE-*StPHR1-#1*, and OE-*StPHR1-#5* roots under normal conditions (**Figure 6A**). However, after aluminum and low phosphorus stress treatments, the inorganic phosphorus content of Col-0, OE-St*PHR1-#1*, and OE-*StPHR1-#5* roots significantly increased. The inorganic phosphorus content of OE-*StPHR1-#1* and OE-*StPHR1-#5* roots were approximately 43% higher than that of Col-0 (**Figure 6A**). Furthermore, longitudinal comparisons within the same plants revealed no significant change in the phosphorus content in the above-ground parts of Col-0, OE-*StPHR1-#1*, and OE-*StPHR1-#5* before and after aluminum toxicity and low phosphorus stress treatments (**Figure 6B**).

Additionally, this study examined the aluminum content in the roots, stems, and leaves of Col-0, OE-*StPHR1-#1*, and OE-*StPHR1-#5* under aluminum ion and low phosphorus stress treatments. The results showed a significant difference in aluminum content between the roots of Col-0 and those of OE-*StPHR1-#1* and OE-*StPHR1-#5* after low phosphorus and aluminum ion stress treatments, while no significant difference was observed in aluminum content among the three genotypes in stems and leaves (**Figure 6C**). In conclusion, these findings suggest that exposure to aluminum toxicity and phosphorus deficiency stress leads to a significant increase in inorganic phosphorus content and a significant decrease in aluminum content in the roots of plants overexpressing the *StPHR1* gene. These results imply that variations in inorganic phosphorus distribution and concentration may contribute to the observed growth phenotypes under aluminum toxicity conditions.

### 3.7 Overexpression of *StPHR1* enhances *Arabidopsis* tolerance to aluminum toxicity and reduces aluminum content in roots

**Figure 7:**
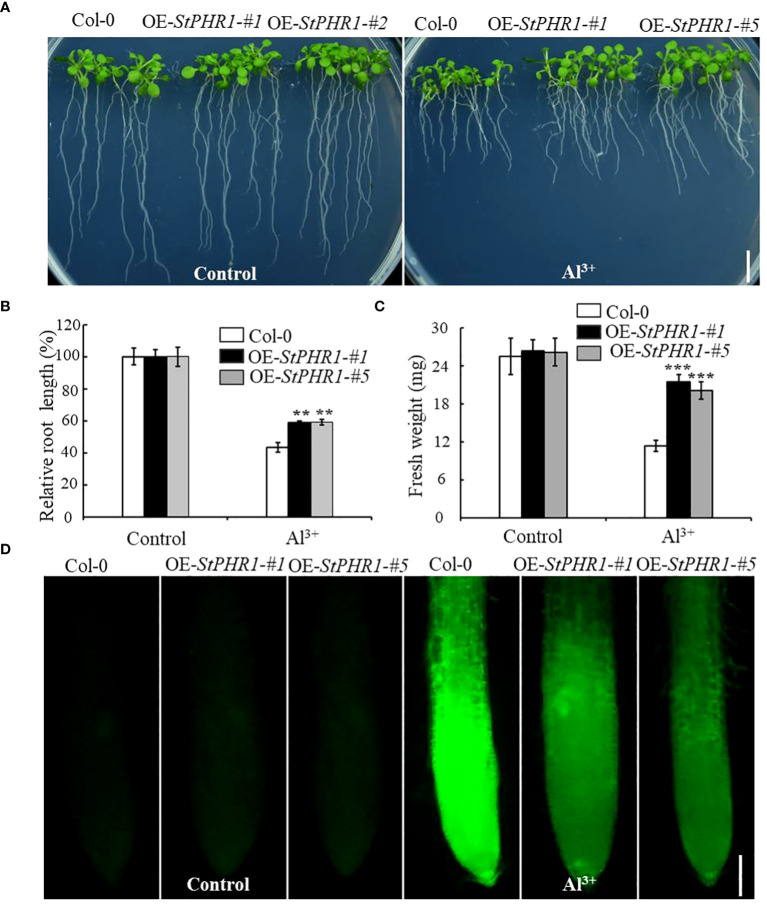
Col-0, OE-*StPHR1-#1*, and OE*-StPHR1-#5* with and without aluminum stress. **(A)** Growth phenotypes, biomass root length **(B)** and fresh weight **(C)**. Scale bar = 1.0 cm. Statistical results of the data. ** indicates significant difference P<0.01, *** represents P<0.001; **(D)** Aluminum staining of root cytoplasm under aluminum stress, Morin staining results of root tips before and after treatment with 250 µM Al^3+^ for 6 h in Col-0, OE-*StPHR1-#1*, and OE*-StPHR1-#5*. Scale bar = 100 μm.

To further elucidate the mechanism underlying the action of *StPHR1* in aluminum toxicity, stress treatments were conducted on Col-0, OE-*StPHR1-#1*, and OE-*StPHR1-#5* with and without the presence of aluminum ions. The results demonstrated that OE-*StPHR1-#1* and OE-*StPHR1-#5* exhibited growth phenotypes that were resistant to aluminum toxicity compared to the Col-0 (**Figure 7A**). This observation was further supported by biomass analysis (**Figures 7B, C**). To validate these findings, Col-0, OE-*StPHR1-#1*, and OE-*StPHR1-#5* plants, both with and without aluminum ion treatment, were stained using the aluminum fluorescent reagent Morin and observed under a fluorescence microscope. The results revealed a significant reduction of aluminum content in the roots of OE-*StPHR1-#1* and OE-*StPHR1-#5* compared to Col-0 (**Figure 7D**). These findings provide strong evidence that the overexpression of *StPHR*1 enhances plant tolerance to aluminum toxicity and reduces the accumulation of aluminum in roots. This offers valuable insight into the balancing mechanism of *StPHR1* in mitigating the dual stresses of phosphorus deficiency and aluminum toxicity.

### 3.8 Analyzing the expression of aluminum-related genes in OE-*StPHR1* and potato species under conditions of aluminum toxicity and phosphorus deficiency

There are numerous genes in *Arabidopsis* that are associated with aluminum stress response and phosphorus uptake response (Maruyama et al., 2019; Barros et al., 2020). Alongside the gene encoding the malate efflux transporter, *AtALMT1*, there are other relevant genes such as the positively regulated transcription factor, *AtSTOP1* (Sensitive to proton rhizotoxicity 1), and the negatively regulated factor, *AtWRKY46*. Additionally, genes involved in aluminum transport in plants include *AtALS1* (Al-sensitive 1), *AtALS3*, and *AtSTAR1* (sensitive to Al rhizotoxicity 1), which act as regulators for *AtALS3* (Larsen et al., 1997; Hoekenga et al., 2006; Larsen et al., 2007; Liu et al., 2009; Ding et al., 2013; Zhang et al., 2018). Thus, in this study, RT-qPCR was employed to examine the expression changes of *AtALMT1*, *AtSTOP1*, *AtWRKY46*, *AtSTAR1*, *AtALS1*, *AtALS3*, and their corresponding homologous genes in potato (*StALMT6*, *StALMT10*, *StSTOP1*, *StWRKY30*, *StSTAR1*, *StALS1*, *StALS3*) under the dual stress conditions of phosphorus deficiency and aluminum toxicity.

**Figure 8:**
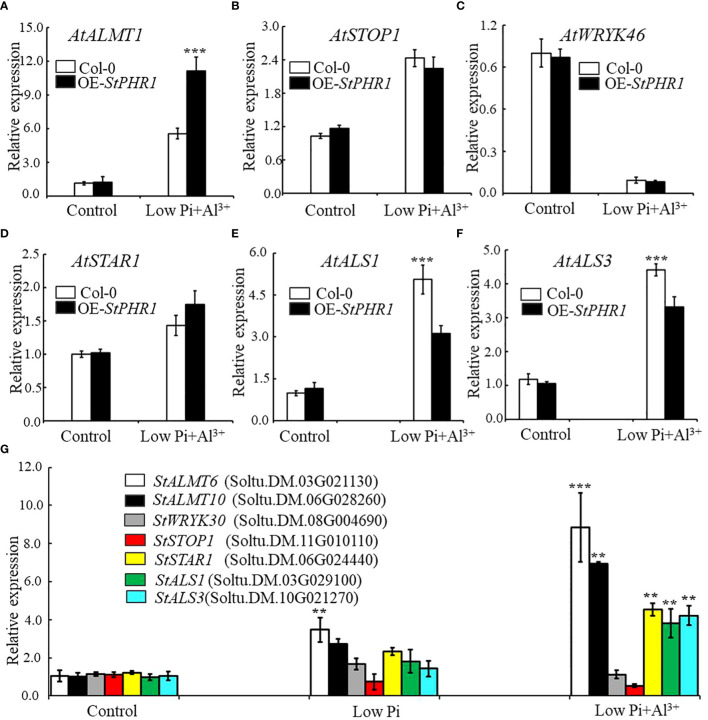
Aluminum-related gene expression changes in Col-0, OE-*StPHR1*, and potato under aluminum toxicity and low phosphorus stress. The relative expression of **(A)** *AtALMT1*, **(B)** *AtSTOP1*, **(C)** *AtWRKY46*, **(D)** *AtSTAR1*, **(E)** *AtALS1*, and **(F)** *AtALS1* genes in Col-0 and OE-*StPHR1* after aluminum ion and low phosphorus treatment. *ACTIN2* was used as an internal standard; **(G)** The relative expression of *StALMT6* (Soltu.DM.03G021130), *StALMT10* (Soltu.DM.06G028260), *StSTOP1* (Soltu.DM.11G010110), *StWRKY30* (Soltu.DM.08G004690), *StSTAR1* (Soltu.DM.06G024440), *StALS1* (Soltu.DM.03G029100), and *StALS3* (Soltu.DM.10G021270) genes in potato roots was assessed after a 12-hour treatment with three different environments: normal, low phosphorus, aluminum ions, and low phosphorus. *ACTIN* (Soltu.DM.03G011750.2) was used as an internal standard. Statistical results of the data. ** indicates significant difference P<0.01, *** represents P<0.001.

The results revealed that the expression of the *AtALMT1* gene was up-regulated in both Col-0 and OE-*StPHR1*. In OE-*StPHR1*, the expression of *AtALMT1* increased to approximately 11.3-fold under normal conditions, while in Col-0, it was nearly 50% lower than that in OE-*StPHR1*, about 5.56-fold (**Figure 8A**). Similarly, the expression of *AtSTOP1* in OE-*StPHR1* was higher than that in Col-0 after aluminum stress treatment, although the difference was not statistically significant (**Figure 8B**). The expression of *AtWRKY46*, which plays a negative regulatory role, decreased in both OE-*StPHR1* and Col-0 (**Figure 8C**). Genes related to aluminum transport, such as *AtSTAR1*, *AtALS3*, and *AtALS1*, exhibited up-regulated expression, but the change in *AtSTAR1* did not reach statistical significance (**Figure 8D**). Furthermore, the expression levels of *AtALS1* and *AtALS3* in OE-*StPHR1* were significantly lower than those in Col-0 (**Figures 8E, F**). Similar results were obtained in potato, where the expression of *StALMT6*, *StALMT10*, *StSTAR1*, *StALS1*, and *StALS3* increased under conditions of low phosphorus and aluminum toxicity treatments. Notably, the increase in *StALMT6* expression was particularly significant (**Figure 8G**). Based on these findings, we propose that the resistance of OE-*StPHR1* to aluminum toxicity and phosphorus deficiency in terms of growth phenotype may be associated with reduced malate efflux detoxification.

### 3.9 The interaction of *StPHR1* and *StALMT6* enhances the role of plants in aluminum toxicity and phosphorus deficiency resistance

In *Arabidopsis*, *ALMT1* encodes a malate efflux transporter located on the plasma membrane, which plays a pivotal role in secreting malate to chelate Al^3+^ and aid in plant response to aluminum toxicity. Aluminum stress induces the up-regulation of *ALMT1* expression (Hoekenga et al., 2006). Additionally, *ALMT1* also plays a crucial role in inhibiting primary root growth and facilitating root structural remodeling during plant response to low phosphorus availability (Wang et al., 2019; Liu et al., 2023). Under low phosphorus stress, *ALMT1* expression is up-regulated, leading to the secretion of malate into the apoplast, where it forms malate-Fe^3+^ complexes that adhere to the cell wall. This, in conjunction with the accumulation of reactive oxygen species (ROS), collectively contributes to the inhibition of primary root growth (Wang et al., 2019). In a previous study, we observed that the expression of the *ALMT1* gene in OE-*StPHR1* plants was nearly 50% higher than in Col-0 plants after treatment with aluminum ions and low phosphorus (**Figure 8A**). Similar results have been confirmed in potato (**Figure 8G**).

**Figure 9:**
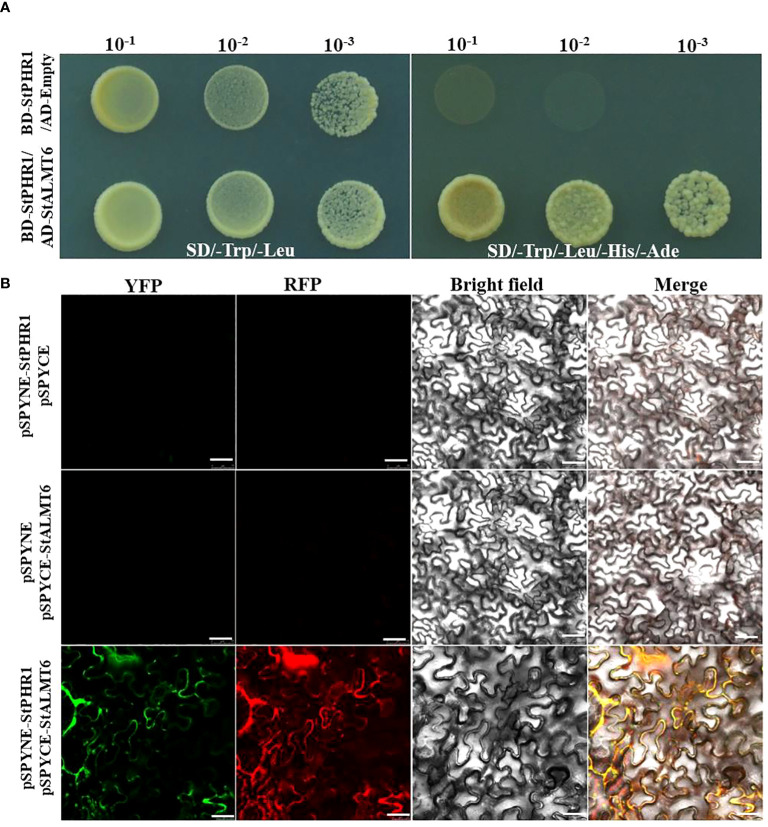
The interaction between potato StPHR1 and StALMT6 was validated *in vitro* and *in vivo*. **(A)** Yeast two-hybrid assay was employed to verify the interaction between StPHR1 and StALMT6; **(B)** Bimolecular fluorescence complementation (BiFC) assay was conducted to confirm the interaction of StPHR1 and StALMT6. The scale bar is set to 50 µm.

To further investigate the potential intergenic interaction between the *StALMT* gene and the *StPHR1* gene in potato, particularly in response to the dual stress of aluminum toxicity and phosphorus deficiency, we constructed yeast two-hybrid and BiFC vectors and performed relevant experiments to validate them. The experimental results clearly demonstrated the interaction between *StPHR1* and *StALMT6* in yeast (**Figure 9A**). Furthermore, the interactions between *StPHR1* and *StALMT6* were confirmed in tobacco through BiFC. Notably, the signal of yellow fluorescent protein was only observed in tobacco leaves co-transformed with *StPHR1*-nYFP and *StALMT6*-cYFP, providing further evidence of the existence of *StPHR1* and *StALMT6* interactions in tobacco (**Figure 9B**). These experimental findings provide compelling evidence supporting the role of *StPHR1* in the dual stress response to aluminum toxicity and phosphorus deficiency. This discovery provides important insights for a deeper understanding of the mechanisms involved in plant adaptation to harsh environments.

## 4 Discussion

In acid soils, the combined action of phosphorus deficiency and aluminum toxicity is a significant limiting factor on crop yields (Chen et al., 2022). This inhibits the growth of primary roots and promotes the growth of lateral roots, which hampers the uptake of water and other nutrients, reduces photosynthesis, and ultimately leads to reduced crop yields (Zheng, 2010; Chen et al., 2022). Transcription factors, which are important regulators of gene expression in plants, play crucial role in various biological processes. Studies have shown that PHR transcription factors are essential for plant response to low phosphorus stress (Bustos et al., 2010). Previous research has identified 15, 16, 12, 18, 35, and 14 PHR transcription factors in *Arabidopsis*, tomato, rice, maize, soybean, and cereal (*Setaria italica*), respectively [ (Bustos et al., 2010; Xue et al., 2017; Xu et al., 2018). In this study, we identified 18 members of the potato *PHR* gene family (**Supplementary Table S1**), which is relatively small compared to the 35 members in soybean. Interestingly, my analysis showed that the differences in the number of *PHR* genes in other species were not significant. It is hypothesized that the number of *PHR* gene family members in plants may be related to genome size and chromosome numbers, while the higher number of *PHR* transcription factors in soybean may be associated with its susceptibility to rhizome production.

Studies of PHR transcription factor families in important crops such as maize, sorghum, and rice, they have been classified them into nine clustering groups. Among these groups, six are shared by all three species, while rice lacks members in the other three groups (Xu et al., 2018). However, in this study, the PHR transcription factor families of Arabidopsis, tomato, and potato were classified into six clustering groups (**Figure 1**). In each cluster group, PHR transcription factors from potato were identified. This finding is consistent with previous research that showed PHR transcription factor members from rice are distributed across all six clustering groups (Xu et al., 2018). Importantly, proteins and genes within the same clustering group share conserved amino acid motifs and gene structures, indicating strong evolutionary and functional conservation (**Figure 2**; **Supplementary Figure S1**). The evolutionary tree reveals that each branch can be traced back to a common ancestral gene, and the number of members within each branch reflects lineage-specific gene duplications and losses (**Supplementary Figure S2**). Therefore, understanding the origin and evolution of the potato PHR transcription factor family is crucial for guiding future studies.

Currently, research on the molecular mechanisms underlying the dual stress of phosphorus deficiency and aluminum toxicity in plant roots has primarily focused on either aluminum tolerance or phosphorus utilization efficiency, with limited consideration of both phosphorus availability and aluminum toxicity (Zheng et al., 2005; Liang et al., 2013). In this study, we specifically examined the role of the *StPHR1* gene in potato plants in response to both phosphorus deficiency and aluminum toxicity. Through genetic evolutionary analysis, we discovered a high degree of homology between the PHR1 proteins of potato, Arabidopsis, and tomato. The MYB and coiled-coil (CC) sequence regions of the PHR1 proteins remained conserved across these three species, indicating the stability of PHR1 during evolution (**Supplementary Figure S1**). Furthermore, we found that the expression level of the *StPHR1* gene was significantly up-regulated in the roots of potato plants under conditions of phosphorus deficiency and aluminum toxicity stress (**Figure 4**). These findings provide valuable insights for further elucidating the functional role of the *StPHR1* gene in response to phosphorus deficiency and aluminum toxicity in potato.

The *AtALMT1* gene encodes a malate efflux transporter localized in the cytoplasmic membrane of Arabidopsis, playing a crucial role in the secretion of malate to sequester Al^3+^ and respond to aluminum toxicity (Hoekenga et al., 2006; Kobayashi et al., 2007; Wang et al., 2022). It has been observed that aluminum stress can induce the up-regulation of *AtALMT1* expression. In this study, gene expression was quantified using q-PCR, revealing that the expression of the *AtALMT1* gene in heterologous overexpressing *Arabidopsis* plants (OE-*StPHR1*), increased by approximately 50% following treatment with low phosphorus plus aluminum ions (**Figure 8A**). Similarly, the expression of the *StALMT6* gene in potato also rose after exposure to low phosphorus plus aluminum ion stress (**Figure 8G**). Based on these findings, we tentatively hypothesized that the phenomena observed in the heterologous overexpression of *Arabidopsis* plants OE-*StPHR1-#1* and OE-*StPHR1-#1*, including improved tolerance to aluminum toxicity, phosphorus-deficient growth phenotypes, and reduced intracellular aluminum content, might be associated with a significant increase in malate exocytosis. To enhance the accuracy of the experimental results, the exocytosis of malic acid can be further analyzed using HPLC in future investigations.

In addition to its role in the response to aluminum stress, *ALMT1* also plays a crucial role in the plant’s response to low phosphorus by inhibiting primary root growth and facilitating root architecture remodeling, and its expression is up-regulated under conditions of low phosphorus (Delhaize et al., 2009; Liu et al., 2023). To explore the potential inter-genic interaction between malate efflux genes and StPHR1 in potatoes, yeast two-hybrid and BiFC assays were conducted in this study to validate the interaction between *StALMT6* and *StPHR1 in vitro* and *in vivo* (**Figure 9**). In future studies, it would be valuable to obtain single and double mutants of the *StALMT6* and *StPHR1* genes using CRISPR/Cas9 technology to further confirm the relevance of *StALMT6* and *StPHR1* in the potato’s response to aluminum toxicity and phosphorus deficiency from a molecular genetic perspective.

## 5 Conclusion

In summary, the study demonstrated the significant role of the *StPHR1* gene in potato plants facing phosphorus deficiency and aluminum toxicity stress. The gene was found to be highly conserved in other plant species and localized in the nucleus of potato cells. Overexpression of the *StPHR1* gene in *Arabidopsis thaliana* resulted in improved resistance of the root system to low phosphorus and high aluminum toxicity. Moreover, these overexpressed plants exhibited lower aluminum content in their roots compared to wild-type plants, indicating the *StPHR1* gene’s regulatory role in reducing aluminum toxicity. Further research should explore the function and regulatory mechanism of the *StPHR1* gene in other crops and investigate its potential application in enhancing plant tolerance to phosphorus deficiency and aluminum toxicity.

## Data availability statement

The original contributions presented in the study are included in the article/**Supplementary Material**. Further inquiries can be directed to the corresponding author.

## Author contributions

FZ: Data curation, Investigation, Methodology, Resources, Writing – original draft, Writing – review & editing, Conceptualization, Formal analysis, Funding acquisition, Software. WW: Formal analysis, Investigation, Writing – review & editing. AY: Investigation, Methodology, Writing – review & editing. QL: Writing – original draft. MC: Formal analysis, Methodology, Writing – original draft. SJ: Funding acquisition, Investigation, Writing – review & editing. YA: Data curation, Formal analysis, Writing – original draft, Writing – review & editing.

## Supplementary material

Supplementary Figure 1 The comparative analysis of the full-length amino acid sequences of PHR1 proteins from potato, tomato, and *Arabidopsis*.

Supplementary Figure 2 Spatial structure modeling of StPHR1, SlPHR1 and AtPHR1.

Supplementary Figure 3 The PCR identified *StPHR1* heterozygous overexpression strains.
