# Supplementary material for: Investigating the involvement of potato (Solanum tuberosum L.) StPHR1 gene in the combined stress response to phosphorus deficiency and aluminum toxicity
Source: Front Plant Sci. 2024 Jun 21;15:1413755. doi: 10.3389/fpls.2024.1413755 (PMC11225713; doi:10.3389/fpls.2024.1413755)
Supplement: Supplementary file 1 [file Table_1.docx]

**Table 1** Molecular characteristic of *StPHR* genes in potato.

| **Gene name** | **Location start** | **Location end** | | **No.of amino acids** | **Predicted**  **location ^a^** | |  |
| --- | --- | --- | --- | --- | --- | --- | --- |
| Soltu.DM.04G010320 | **chr04**:10816542 | | 10821943 | 319 | | Nucl/ Cyto/ Chlo | |
| Soltu.DM.04G019420 | **chr04**:44520423 | | 44530923 | 475 | | Nucl | |
| Soltu.DM.05G005130 | **chr05**:4458148 | | 4460422 | 407 | | Nucl/ Cyto | |
| Soltu.DM.05G026640 | **chr05**:54664751 | | 54670851 | 472 | | Nucl/ Cyto/ Plas | |
| Soltu.DM.06G001980 | **chr06**:2367066 | | 2378516 | 456 | | Nucl/ Chlo | |
| Soltu.DM.06G019150 | **chr06**:45871013 | | 45873285 | 243 | | Nucl/ Cyto | |
| Soltu.DM.08G021740 | **chr08**:51138691 | | 51149660 | 712 | | Chlo/ Nucl | |
| Soltu.DM.09G022120 | **chr09**:57595928 | | 57606229 | 450 | | Nucl/ Chlo/ Cyto | |
| Soltu.DM.09G028940 | **chr09**:64951516 | | 64956644 | 289 | | Nucl/ Cyto | |
| Soltu.DM.09G007960 | **chr09**:8281882 | | 8284064 | 256 | | Nucl/ Cyto | |
| Soltu.DM.10G021990 | **chr10**:54146475 | | 54149411 | 500 | | Nucl/ Chlo | |
| Soltu.DM.10G024990 | **chr10**:56634517 | | 56637418 | 415 | | Chlo/ Nucl | |
| Soltu.DM.10G026950 | **chr10**:58282691 | | 58284760 | 374 | | Nucl/ Cyto/ Chlo | |
| Soltu.DM.10G029270 | **chr10**:60052336 | | 60055793 | 402 | | Nucl | |
| Soltu.DM.11G012550 | **chr11**:15381502 | | 15387679 | 306 | | Nucl/ Chlo | |
| Soltu.DM.11G021700 | **chr11**:41738746 | | 41745129 | 324 | | Nucl/ Chlo | |
| Soltu.DM.12G002760 | **chr12**:2223943 | | 2228095 | 300 | | Nucl | |
| Soltu.DM.12G020640 | **chr12**:48955643 | | 48961900 | 377 | | Nucl/ Chlo/ Cyto | |
