# Supplementary material for: Investigating the involvement of potato (Solanum tuberosum L.) StPHR1 gene in the combined stress response to phosphorus deficiency and aluminum toxicity
Source: Front Plant Sci. 2024 Jun 21;15:1413755. doi: 10.3389/fpls.2024.1413755 (PMC11225713; doi:10.3389/fpls.2024.1413755)
Supplement: Supplementary file 2 [file Table_2.docx]

**Table S2.** Primers used in this study

| **Primers** | **Sequence 5′-3′** | **Purpose** |
| --- | --- | --- |
| *St-Actin-F* | GGGATGGAGAAGTTTGGTGGTGG | RT-qPCR |
| *St-Actin-R* | CTTCGACCAAGGGATGGTGTAGC |  |
| *StPHR1-F1* | GCAGCAACCTGCCAATCTTC |  |
| *StPHR1-R1* | CAACTGCAAGGCTTTCGACC |  |
| *StLPR1-F* | TGATGCCTGGACATGTTACAA |  |
| *StLPR1-R* | TGCCGGCAACAGACTCTTCT |  |
| *StNLA-F* | CACGAAGCGTTGTTGGTCTG |  |
| *StNLA-R* | AGCAATTTCTGCGCACGTTT |  |
| *StPHO1-F* | TTGGGTGGAGACAGTGATGC |  |
| *StPHO1-R* | GCGGAATGGCAATGGAACTG |  |
| *StPHT1;1-F* | TCCAAACGCCACAACATTCG |  |
| *StPHT1;1-R* | CGCAACCAAGGACGATCAAC |  |
| *StPHT1;4-F* | GGTGCTATACCGGCTTTGCT |  |
| *StPHT1;4-R* | ATGCAGCCCATGACGATGAA |  |
| *StWRKY45 -F* | AGCATCATCGTCGTCATCTCA |  |
| *StWRKY45 -R* | TTGGCTCCTTGTTTGGAAAGC |  |
| *StALMT6-F* | TCAGTGGTGCACTACGACAC |  |
| *StALMT6-R* | AGGATCTCCGGGGCTTAGTT |  |
| *StALMT10-F* | ACACGGTTCGATCAAGGCAA |  |
| *StALMT10-R* | GTGAGGATGGCCCAAATGGA |  |
| *StSTOP1-F* | CAGTGTTGCGAAGGTGGAAG |  |
| *StSTOP1-R* | TGAGTGTGTGGTGCAAGGAT |  |
| *StWRKY30-F* | TGACCCAGCAGCATGTCATT |  |
| *StWRKY30-R* | GCTGCTGCTTGAGACTGAGA |  |
| *StSTAR1-F* | GAGTTGCACTTGCCAGAACC |  |
| *StSTAR1-R* | TCTCCCCATCAACCAACAGG |  |
| *StALS1-F* | CACTTGCAAAGCCTGAAGCA |  |
| *StALS1-R* | CTGTGCAAACAGAACCGACG |  |
| *StALS3-F* | TTATTCCTGTCGCGGGGATG |  |
| *StALS3-R* | TAGCCCAGTCATTGCACCTG |  |
| *At-Actin2-F* | ATTCAGATGCCCAGAAGTCTTGTTCC |  |
| *At-Actin2-R* | ACCACCGATCCAGACACTGTACTTCC |  |
| *AtALMT1-F* | ACTTGAGAGAGCTGAGTGACC |  |
| *AtALMT1-R* | TCTTCTCGGGTCTTCATTCCC |  |
| *AtSTOP1-F* | CCAAGTTCCATCTCAAGCTTTTCT |  |
| *AtSTOP1-R* | TGGGACGTAAAACCTGCGAA |  |
| *AtWRKY46-F* | ACATCACATCCCCGAAGACG |  |
| *AtWRKY46-R* | ACTTCTTCGGACTTGGTCGG |  |
| *AtSTAR1-F* | GCCATGCCATCACTTTGGTC |  |
| *AtSTAR1-R* | AGATCCATCATCGGCGACTC |  |
| *AtALS1-F* | CAATCGCCGGAATGTTGGTC |  |
| *AtALS1-R* | TTGCAACGTCGCTTGTCTTG |  |
| *AtALS3-F* | TGAGCAGCAAACGGAATCCT |  |
| *AtALS3-R* | CTCGCAAAGCCGTGCATATC |  |
| *St-PHR1-F2* | CATGGGTCTCACAACATGGAGGCGCGTCCTGCTTTATC | Subcellular localization |
| *St-PHR1-R2* | CATGGGTCTCATACATTCATCGACCTTGGCACGTTTCATTGG |  |
| *St-PHR1-F3* | ATGCATGAATGGCAGGAGGCAATGCCCTATCT | Vector construction |
| *St-PHR1-R3* | GACTAGTCTACAAGTTAGCTGCTTCTCCTCTGC |  |
| *St-PHR1-F4* | TCAGCATAGGTGCAACGCTT | Identification of overexpressed plants |
| *St-PHR1-R4* | CCATGACCATGAAAGCGCAA |  |
