# Supplementary figures and images for: Investigating the involvement of potato (Solanum tuberosum L.) StPHR1 gene in the combined stress response to phosphorus deficiency and aluminum toxicity
Source: Front Plant Sci. 2024 Jun 21;15:1413755. doi: 10.3389/fpls.2024.1413755 (PMC11225713; doi:10.3389/fpls.2024.1413755)

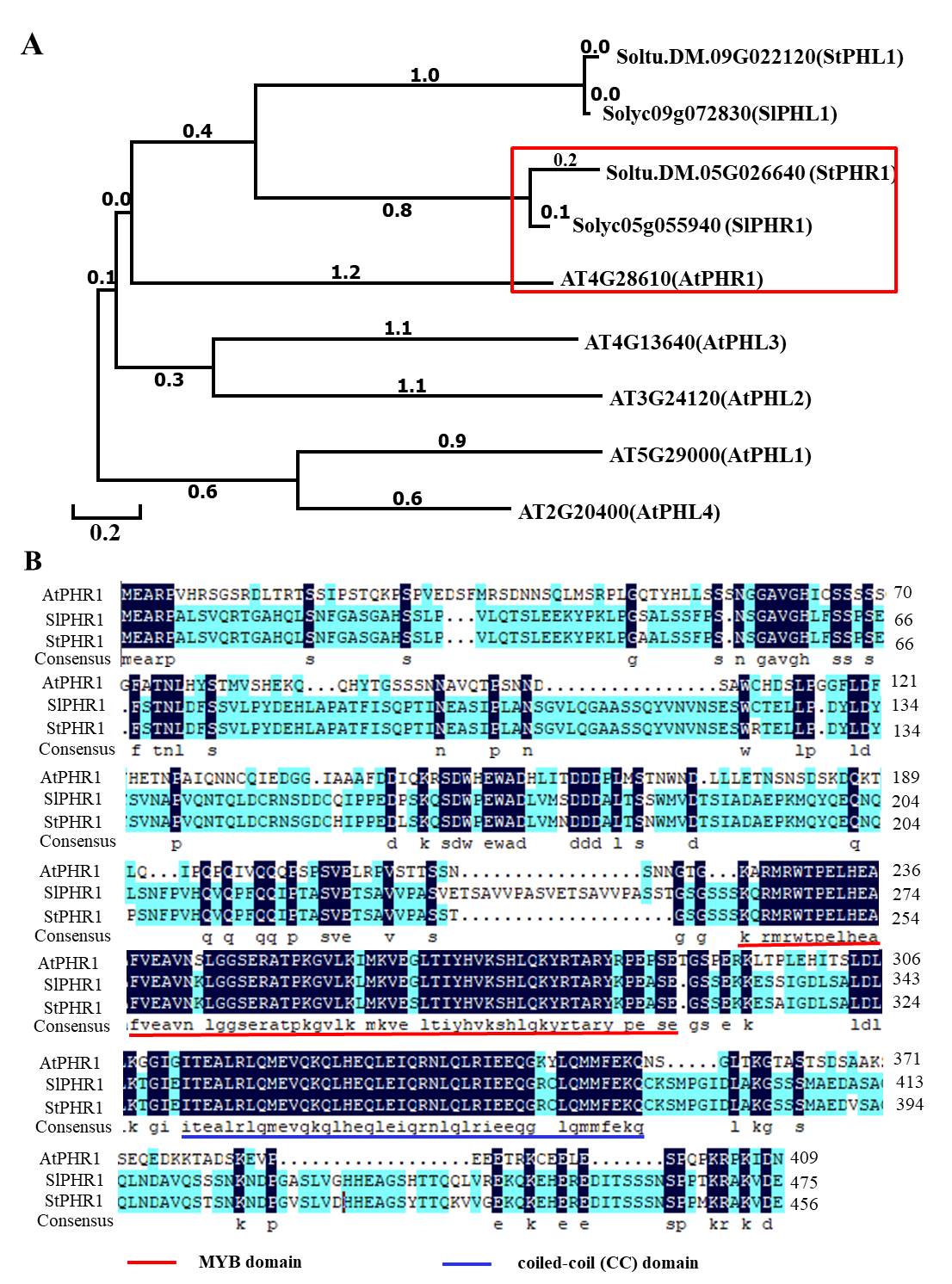

Supplement: Supplementary Figure 1 — The comparative analysis of the full-length amino acid sequences of PHR1 proteins from potato, tomato, and Arabidopsis. [file Image_1.jpeg]

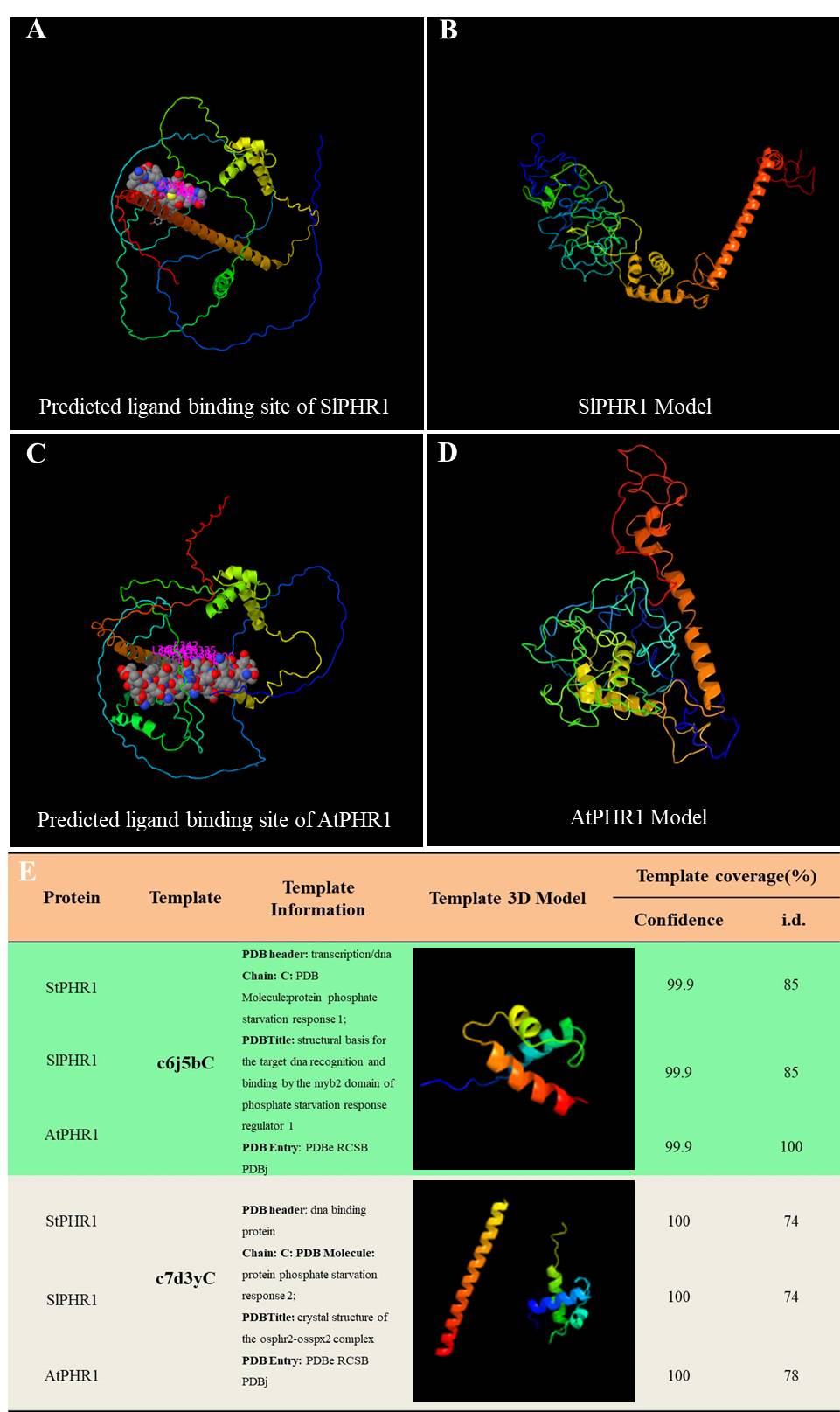

Supplement: Supplementary Figure 2 — Spatial structure modeling of StPHR1, SlPHR1 and AtPHR1. [file Image_2.jpeg]

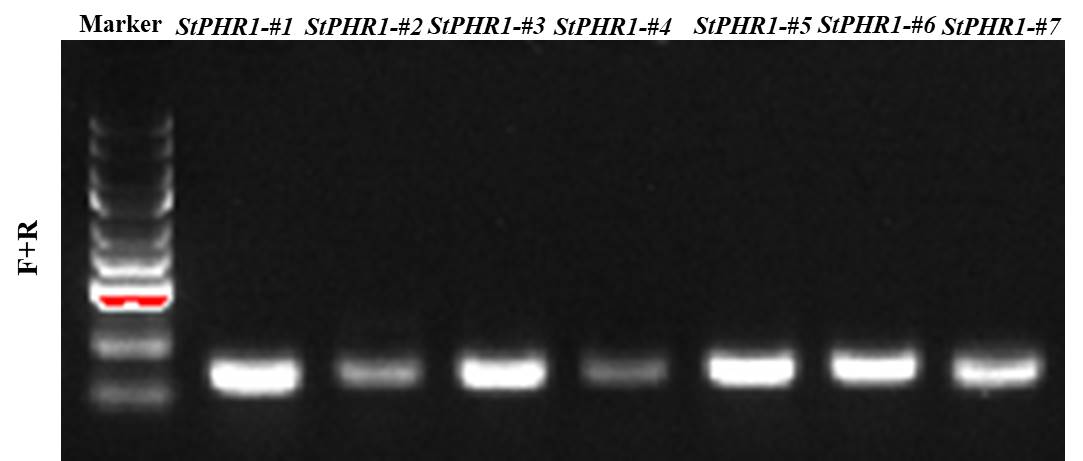

Supplement: Supplementary Figure 3 — The PCR identified StPHR1 heterozygous overexpression strains. [file Image_3.jpeg]
